# Pattern of Prescribing Proton Pump Inhibitors: Evaluating Appropriateness and Factors Contributing to Their Adverse Effect Reaction Risk

**DOI:** 10.3390/jcm13206187

**Published:** 2024-10-17

**Authors:** Aymen A. Alqurain, Mohammed F. Alomar, Shatha Fakhreddin, Zahrah Julayh, Zahra Korikeesh, Samaher Al-Shaibi, Afnan Alshnbari, Alaa Al Helaili, Luma Ameer, Manal Surour, Sherihan Ghosn, Dania Hussein, Bader AlAlwan, Fadhel A. Alomar, Keshore R. Bidasee

**Affiliations:** 1 Department of Clinical Practice, Faculty of Pharmacy, Northern Border University, Rafha 91911, Saudi Arabia; 2 College of Medicine, Imam Abdulrahman Bin Faisal University, Dammam 31441, Saudi Arabia; 2190005716@iau.edu.sa; 3 Department of Pharmacy, Mohammed Al-Mana College for Medical Sciences, Dammam 34222, Saudi Arabia; 2021201450@machs.edu.sa (S.F.);; 4 Foundation Year Department, Mohammed Al-Mana College for Medical Sciences, Dammam 34222, Saudi Arabia; 5 Department of Pharmacology, College of Clinical Pharmacy, Imam Abdulrahman Bin Faisal University, Dammam 31441, Saudi Arabia; dahussein@iau.edu.sa; 6 Department of Medical Laboratory Sciences, Mohammed Al-Mana College for Medical Sciences, Dammam 34222, Saudi Arabia; 7 Departments of Pharmacology and Experimental Neuroscience, University of Nebraska Medical Center, Omaha, NE 68198, USA; 8 Nebraska Redox Biology Center, Lincoln, NE 68503, USA

**Keywords:** proton pump inhibitors, adverse drug reactions, pattern of prescribing, dose intensity, medical care medicine

## Abstract

**Background/Objectives**: Proton pump inhibitors (PPIs) are amongst the most commonly prescribed classes of medication. However, inappropriate PPI use can lead to several adverse drug reactions (ADRs). Limited data exist on factors contributing to the risk of ADRs associated with PPI prescribing patterns in the Eastern Region of Saudi Arabia. This retrospective, cross-sectional study aimed to assess the prevalence and the pattern of PPI use and to identify factors contributing to the risk of ADRs. **Methods**: Data were collected from electronic medical records of patients at Al-Qateef Central Hospital from January 2020 to December 2021. The inclusion criteria included patients aged ≥40 years attending an outpatient medical care clinic. PPI prescribing patterns were categorized based on their dosage intensity into low-dose, medium-dose (MD), and high-dose (HD) categories. Binary and multinominal logistic regression models were used to determine the relationship between PPI prescribing patterns and use, categorized by MD or HD, and patient characteristics, adjusted for significant covariates. Results are presented as adjusted odds ratio (OR) with corresponding 95% confidence intervals (95% CI). **Results**: The study included 41,084 patients. The prevalence of PPI prescribing was 31%. PPI users were more frequently found to be females than males (52% vs. 50%, *p* = 0.013); they were also likely to be prescribed more medications (7 vs. 6, *p* < 0.001), but less likely to have gastritis-related diseases (34% vs. 32%, *p* < 0.001) compared to non-users. PPI HD users were more likely male (56% vs. 43%, *p* < 0.001), older (53 vs. 52 years, *p* < 0.001), and prescribed more medications (11.8 vs. 2.8, *p* < 0.001) compared to MD users. PPI usage was associated with concurrent use of antiplatelet drugs (OR = 1.08, 95% CI 1.01–1.15). An increasing number of prescribed medications was associated with HD usage (OR = 1.13, 95% CI 1.12–1.14), but negatively associated with MD usage (OR = 0.7 95% CI 0.69–0.71). Female gender was negatively associated with HD usage (OR = 0.85, 95% CI 0.79–0.91). **Conclusions**: Our findings indicate that 31% of the included cohort were prescribed PPI. Inappropriate PPI prescribing related to the drug’s omission is a concern as PPI non-users presented with valid indications such as gastritis. Male gender and increasing NPM were the common factors contributing to increased risk of PPI ADR. This study points to the importance of re-evaluating PPI use to ensure effective therapy with minimum risks of ADR.

## 1. Introduction

Proton pump inhibitors (PPIs) are a class of highly effective medications, used for the treatment of a wide range of gastrointestinal disorders. PPI drugs are currently recommended as the first-line treatment for peptic ulcer disease (PUD), Zollinger–Ellison syndrome, gastroesophageal reflux disease (GERD), and erosive esophagitis. They are also an essential component of the triple therapy regimen used to treat Helicobacter pylori infections [1,2]. Additionally, PPI drugs are commonly used as prophylaxes to prevent the incidence of drug-induced peptic ulcer disease, attributed to the use of medications such as nonsteroidal anti-inflammatory drugs (NSAIDs). PPI use has also been found to reduce the risk of gastrointestinal bleeding associated with antiplatelet drugs [3,4,5]. Despite its widespread use, recent studies have raised concerns regarding the long-term safety profile of PPI, highlighting the need for a critical examination of associated risks [6,7].

Therapeutic guidelines recommend a medium-intensity PPI dose for the symptomatic relief of uncomplicated GERD for a limited duration of eight weeks. If symptoms are not alleviated, a high-intensity PPI dose is recommended until symptoms are controlled [8]. Periodic reassessment is recommended to encourage reducing the dose to the minimum effective concentration once symptoms are controlled. Prophylactic PPI use at lower doses may also be suitable for elderly patients at higher risk of developing PUD. The dose intensities of commonly used PPI drugs are shown in Table 1 [8,9].

Clinical evidence has shown that long-term PPI use is associated with nutritional deficiencies as a result of their ability to impair the absorption of various nutrients such as calcium, magnesium, iron, and vitamin B12 [3]. Several observational studies have consistently demonstrated an association between PPI use and an increased risk of osteoporosis, ultimately leading to increased susceptibility to bone fractures [3,7,10]. This risk is particularly evident in elderly populations, who experience accelerated bone loss due to aging, and are more vulnerable to fractures.

In addition to the increased risk of bone fractures, the use of PPIs has been linked to a range of additional adverse effects, including headache and migraine, cognitive impairment, dementia, chronic kidney disease (CKD), cardiovascular disorders (such as myocardial infarction, atrial arrhythmia, and atrial hypertension), and gastrointestinal malignancies [11,12,13].

PPIs have also been associated with an increased risk of infections, including community-acquired pneumonia and gastrointestinal infections, due to their hypochlorhydria effect [3]. Chronic kidney disease (CKD) has emerged as another potential safety concern associated with long-term PPI use [3,14]. Studies have shown that PPIs may increase the risk of CKD by 17–50%, with an absolute annual excess risk of 1.8% compared to histamine 2 antagonist [15,16].

Several factors contribute to an increased risk of adverse drug reactions (ADRs), which can be categorized into patient-specific, drug-related, and health system-related factors. Advancing age increases the susceptibility to PPI-induced ADR, as it can alter the pharmacokinetic and pharmacodynamic proprieties of the drugs [17]. Gender based differences are also important; females appear to have a greater tendency of developing ADRs compared to males [18]. Polypharmacy, which is defined as the concurrent intake of five or more medications, may also increase the incidence of ADRs through drug–drug interactions (DDIs) [9,19]. Inappropriate prescribing practices, such as dosage errors or unnecessary prescriptions, further contribute to increased ADR risk, particularly in older patients [20].

Several studies conducted globally have reported overutilization of PPIs [21,22,23,24,25]. PPI drugs are recommended for short-term use, typically for limited periods of time, not exceeding two months [26]. Such regimens often encompass short-course PPI therapy for dyspepsia, PUD, and GERD, but often PPIs may be prescribed for a variety of reasons. A study by Ladd et al. reveals that 76% of hospitalized patients receiving PPI therapy did not have an appropriate indication to warrant their use [21]. Similarly, Chia et al. found that 54.1% of hospitalized patients receiving a PPI did not meet FDA-approved indications for PPIs [22]. A qualitative study conducted in Saudi Arabia also reported that PPI drugs were often prescribed inappropriately to manage nausea and vomiting rather than acid-related diseases [27].

The current study aims to empower evidence-based decision-making regarding safe prescribing practices for PPIs. To achieve this aim, this study was designed to determine the prevalence and prescribing patterns associated with the use of PPIs. Furthermore, this study aims to identify patient-specific characteristics among PPI users, which may potentiate the risk of PPI-induced ADRs, and to determine the association between the identified patients’ characteristics and prescribing patterns. The findings of this study ultimately aim to influence current clinical guidelines by promoting a more appropriate utilization of PPIs to improve patient outcomes.

## 2. Materials and Methods

### 2.1. Study Design and Data Source

This retrospective, cross-sectional observational study utilized data collected from the electronic medical records of patients presented to Al-Qatif Central Hospital (QCH) between 1 January 2020 and 31 December 2021. QCH is a 360-bed tertiary referral hospital, and is one of the largest public hospitals in the Eastern Region of Saudi Arabia serving Al-Qatif city and offers a comprehensive range of essential clinical care services: medical, surgical, emergency, and mental health. The inclusion criteria included patients aged 40 years or older attending outpatient medical care clinics during the study period. The exclusion criteria included patients younger than 40 years attending surgical, dental, or gynecology/obstetric care clinics or the emergency department, and those admitted to inpatient wards or intensive care units. The age of 40 years was set as an inclusion criterion to assess the impact of aging on the epidemiology of multiple morbidities. This approach facilitates an examination of how changes in comorbidities and co-prescribed medications may influence the prescribing patterns of PPIs [28]. For patients with multiple visits within the study timeframe, only data from the first reported visit were included within this study.

### 2.2. Ethical Consideration, Data Collection, Measures and Definitions

This study was approved by the Institutional Review Board (IRB) committee at Mohammed Al-Mana College for Medical Sciences (SR/RP/79) and the IRB committee at QCH (QCH-SREC019/2022). Patients’ demographic data, comorbidities, and laboratory findings were collected from their medical electronic records, while medication details were verified using pharmacy electronic records.

Patient comorbidities were identified based on information reported in the medical record and by applying the Rx–risk comorbidities index to the prescribed medication list. Comorbidities were coded as per the International Classification of Disease, 10th revision 2016 (ICD-10) [29,30]. The Charlson comorbidity index (CCI) was calculated to predict the one-year mortality risk [31]. Creatinine clearance (CrCl) was calculated using the Cockcroft–Gault Equation [32]. Due to a lack of full discrimination of specific morbidities, asthma and chronic obstructive pulmonary disease (COPD) were consolidated under one variable “respiratory diseases”, while osteoarthritis and rheumatoid arthritis were consolidated under “arthritis-related diseases”. All types of anemias were consolidated under “anemia” and different types of pain in the lower and upper back, muscles, bones, joints, ligaments, and tendons were consolidated under “musculoskeletal pain”. Peptic ulcer, gastroesophageal reflux disease, or any other form of gastritis disorder were consolidated under “gastritis-related diseases” because of overlapping clinical symptoms and treatment.

Collected data on prescribed and dispensed medications, including long-term, short-term, and as-needed prescriptions and supplements, and the medications, were coded as per the Anatomical Therapeutic Chemical (ATC) classification system [33]. Medications were reported as the first level or the second level order of the ATC system throughout the study. The total number of prescribed medications (NPM) was counted. Long-term medicines were defined as those without a specific duration of use. PPI doses were classified based on the dose intensity into low dose, MD, or HD as described in Table 1 [4]. PPI appropriateness was assessed as previously described by Liu et al. [34].

This study measured the association between PPI prescribing with the occurrence of several factors that were identified to potentiate the drugs’ ADRs. As stated in the introduction, these factors can be categorized into patient-specific, drug-related, and health system-related factors [9,17,18,19,20]. Identifying these associations will help the medical care team evaluate the appropriateness of PPI use and develop care plans to reduce the ADR risk.

In our study, the prescribing pattern of PPIs was assessed under two classifications. First, patients were grouped based on age into middle-aged patients (<65 years) and older patients (≥65 years), to assess the prescribing pattern differences between these two groups. Second, a more detailed classification categorized all patients into six age groups (40–49, 50–59, 60–69, 70–79, 80–89, 90 years or older) to assess the trends of the prescribing pattern.

### 2.3. Statistical Analysis

Demographic variables, comorbidities, and frequency and intensity of the prescribed medications are reported using mean and standard deviation (SD) for parametric continuous variables, median/interquartile range (IQR) for non-parametric continuous variables, and number/frequency (%) for binary variables. Student’s *t*-test and the Mann–Whitney U test were used for comparing continuous variables and non-parametric variables, respectively. The chi-square test was used to compare the frequency of categorical variables between groups. Analysis of variance (ANOVA) was used to identify and compare trends and patterns of PPI prescribing across different age groups. Two types of logistical regression models were used in the current study. Firstly, a binary logistical regression model was used to determine the association between prescribing PPI and patients’ characteristics amongst the entire cohort, middle-aged patients, and older patients. Secondly, a multinomial logistical regression model was used to determine the association between prescribing MD or HD of PPIs and patients’ characteristics amongst the whole cohort, middle-aged patients, and older patients regarding not prescribing PPIs. Both models were adjusted with significant covariates (age, sex, weight, CCI, CrCl, and NPM). Results from these models are presented as adjusted odds ratio (OR) and 95% confidence intervals (95% CI). Covariates were included in models if they reached a statistically significant level at *p* < 0.05 in univariate analyses. Multicollinearity was assessed using the variance inflation factor. Statistical analysis was performed using the Statistical Package for Social Sciences version 26 (SPSS, Inc., Chicago, IL, USA) and *p* ≤ 0.05 was considered statistically significant.

## 3. Results

A total of 41,084 patients were included in the study and the prevalence of PPI prescribing was 31%. Table 2 shows that females were more likely to be prescribed PPI compared to males (52% vs. 50%, *p* = 0.013). Overall, PPI users were prescribed more medications compared to non-users (7 vs. 6, *p* < 0.001). Interestingly, Table 2 reveals that gastritis-related diseases and constipation were more frequently reported among PPI non-users (34% vs. 32%, *p* < 0.001, and 10% vs. 9%, *p* = 0.02, respectively) compared to users. Regarding co-prescribed medications, PPI users were more likely to receive anticoagulants (12% vs. 11%, *p* = 0.03), antiplatelets (13% vs. 12%, *p* = 0.006), and calcium channel blockers (CCB) (10% vs. 9%, *p* = 0.004) compared to PPI non-users (Table 2).

**Figure 1:**
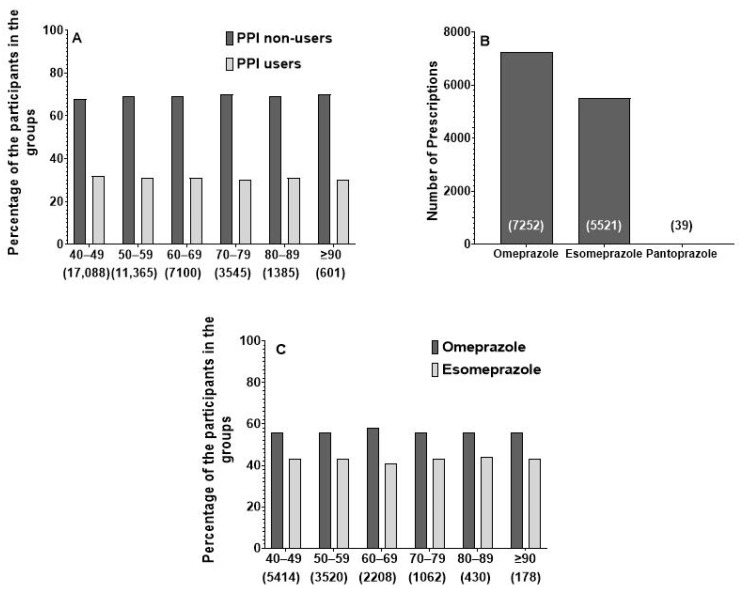
Prescribing pattern of proton pump inhibitors within the entire cohort. Panel (**A**) presents the pattern of proton pump inhibitor drugs prescribed for the entire cohort over different age groups. Panel (**B**) presents the prevalence and pattern of specific proton pump inhibitor drug prescribing. Panel (**C**) presents the overall prescribing pattern of proton pump inhibitors prescribing over different age groups.

When assessing the prescribing patterns of PPIs, Figure 1 shows that omeprazole (*n* = 7252, 56.6% of PPI users) and esomeprazole (*n* = 5521, 43% of PPI users) were the most frequently prescribed. Pantoprazole was reported in only 39 prescriptions (0.4% of PPI users). No significant difference was observed between the age groups in terms of the prevalence of omeprazole vs. esomeprazole.

**Figure 2:**
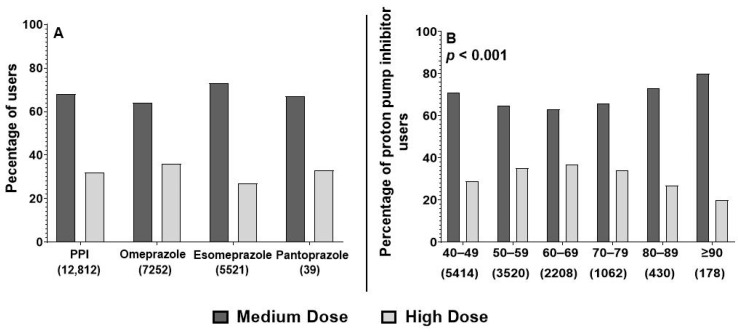
Pattern of high-dose versus medium-dose prescribing among proton pump inhibitors users. Panel (**A**) shows the difference in dose intensity among users of omeprazole, esomeprazole, and pantoprazole. Panel (**B**) shows the difference in dose intensity prescribing across different age groups among proton pump inhibitor users. *p*-value represents the results of univariate analysis used to determine differences in prescribing prevalence between different age groups.

Further analysis was conducted to determine the prescribing patterns based on different dose intensities of PPI. Figure 2 shows that among PPI users, PPI medications were predominantly prescribed at either a MD or HD. Notably, 68% of all PPI prescriptions (*n* = 8683), 73% of esomeprazole (*n* = 4023), 64% of omeprazole (*n* = 4634), and 67% of pantoprazole (*n* = 26) were categorized under MD. Figure 2 also reveals a significant difference in dose intensity prescribing across different age groups. Most patients in the study received a MD of PPI, with the lowest usage observed in the 60–69 years group (63%), which also exhibited the highest proportion of HD PPI usage. The highest usage of MD (80%) was observed among patients aged 90 years and older.

When comparing HD vs. MD users, Table 3 shows that HD users were more frequently male (57% vs. 48%, *p* = 0.013), older (53 vs. 52, *p* < 0.001), and had a higher body weight (81 kg vs. 71 kg, *p* < 0.001) compared to MD users. Interestingly, HD users were prescribed more medications (12 vs. 3, *p* < 0.001) and had a higher CrCl (105 vs. 96, *p* < 0.001) compared to MD users. Regarding morbidities, Table 3 shows that HD users were less likely to have renal diseases (1% vs. 2%, *p* = 0.02) and arthritis-related diseases (69% vs. 71%, *p* = 0.01) compared to MD users. Conversely, MD users were more likely to be prescribed anticoagulants (12% vs. 11%, *p* = 0.02), antiplatelets (12% vs. 11%, *p* = 0.001), CCB (11% vs. 8%, *p* < 0.001) and NSAIDs (49% vs. 47%, *p* = 0.03) compared to HD users (Table 3).

Among PPI users, older patients were more frequently female (55% vs. 51%, *p* < 0.001), and had higher CCI scores (5 vs. 2, *p* < 0.001) and lower CrCl (74 vs. 105, *p* < 0.001), compared to middle-aged patients (Table 4). Table 4 also shows that there was no significant difference between older and middle-aged patients in terms of the pattern of PPI use or the selection of dose intensity. Additionally, among PPI users, middle-aged patients were more likely to have pain (61% vs. 59%, *p* = 0.009) and arthritis-related diseases (71% vs. 69%, *p* = 0.03), but less likely to have gastritis-related diseases (29% vs. 42%, *p* < 0.001) and osteoporosis (18% vs. 24%, *p* < 0.001) compared to older patients. Regarding co-prescribed medications, middle-aged patients within the PPI user group were more likely to be prescribed anticoagulants (12% vs. 11%, *p* = 0.01), antiplatelets (14% vs. 11%, *p* < 0.001), CCB (11% vs. 9%, *p* = 0.001), paracetamol (60% vs. 55%, *p* < 0.001), and tricyclic antidepressants (TCAs, 4% vs. 3%, *p* = 0.04) compared to older patients (Table 4).

After adjusting the regression models for age, sex, weight, CCI, CrCl, and NPM, PPI prescribing was associated with concurrent use of CCB (OR = 1.09, 95% CI 1.01–1.16) and antiplatelets (OR = 1.08, 95% CI 1.01–1.15), but negatively associated with increasing NPM (OR = 0.96, 95% CI 0.95–0.97) in the entire cohort (Table 5). In addition, regression analysis indicated that PPI prescriptions among older patients coincided with associated NSAIDs use (OR = 1.1, 95% CI 1.01–1.2), but was found to be negatively associated with osteoporosis (OR = 0.85, 95% CI 0.75–0.96) and increasing NPM (OR = 0.97, 95% CI 0.96–0.98). Furthermore, Table 5 shows that PPI prescriptions among middle-aged patients were associated with concurrent use of antiplatelets (OR = 1.1, 95% CI 1.03–1.2), and CCB (OR = 1.09, 95% CI 1.01–1.2), but negatively associated with gastritis (OR = 0.92, 95% CI 0.87–0.98) and increasing NPM (OR = 0.96, 95% CI 0.955–0.965).

In this study, the multinomial logistical regression model was utilized to identify factors associated with prescribing MD or HD PPI regimens among the cohort, middle-aged patients, and older patients. The model was adjusted for age, sex, weight, CCI, CrCl, and NPM, with the OR indicating the likelihood of MD or HD prescription with reference to not prescribing PPI. The analysis revealed that MD prescribing was associated with increasing CrCl value (OR = 1.002, 1.001, and 1.004, *p* < 0.05), but negatively associated with increasing NPM (OR = 0.7, 0.7, 0.7, *p* < 0.05) and increasing weight (OR = 0.99, 0.99, 0.99, *p* < 0.05) across the entire cohort, middle-aged patients, and older patients, respectively (Table 6). Notably, among older patients, prescribing MD PPI regimens was associated with SSRI use (OR = 1.98, *p* < 0.5), but negatively associated with pain (OR = 0.88, *p* < 0.05) and osteoporosis (OR = 0.85, *p* < 0.05).

Conversely, several factors were found to be associated with HD use among different groups. HD PPI regimens were found to be associated with increasing NPM (OR = 1.13, 1.13, and 1.12, *p* < 0.05), but negatively associated with being female (OR = 0.85, 0.85, 0.84, *p* < 0.05), having renal diseases (OR = 0.71, 0.71, 0.57, *p* < 0.05), and concurrent use of CCB (OR = 0.85, 0.85, 0.72, *p* < 0.05) or paracetamol (OR = 0.89, 0.89, 0.86, *p* < 0.05) across the entire cohort, middle-aged patients, and older patients, respectively (Table 6).

In addition, Table 6 shows that prescribing HD PPI was negatively associated with the presence of constipation (OR = 0.85, 0.84, *p* < 0.05), arthritis-related diseases (OR = 0.9, 0.91, *p* < 0.05), or concomitant antacid prescriptions (OR = 0.83, 0.83, *p* < 0.05) in the entire cohort and middle-aged patients. Among older patients, HD prescribing was negatively associated with osteoporosis (OR = 0.78, *p* < 0.05) and concurrent use of antiplatelets (OR = 0.68, *p* < 0.05) (Table 6).

## 4. Discussion

PPI drugs are some of the most commonly prescribed medications worldwide, raising legitimate concerns about their associated ADRs with chronic use [35]. This study provides valuable insights into the patterns associated with PPI prescribing and use in the Eastern Region of the Kingdom of Saudi Arabia and identifies the key factors contributing to the risk of PPI ADRs. The first finding from this study reveals that 31% of adults aged ≥40 years in the cohort were prescribed PPIs. This rate is significantly lower than other studies conducted in Saudi Arabia, where the prevalence rate was 62.5%, and is higher than the global prevalence of 23.4% reported in a systematic review conducted in 2023 [36,37]. This discrepancy might be attributed to the differences in patient cohorts, as our study focused on outpatients attending medical clinics, whereas the referenced study primarily investigated hospitalized patients.

The main indication of PPI includes the treatment of gastric acid disorders and to prevent PUD that may be induced by gastric irritant medications, such as NSAIDs. Table 2 shows that 9502 out of 13,563 patients (70%) diagnosed with gastric-related diseases and 13,369 of out 19,504 patients (68%) receiving NSAIDs, were not prescribed PPIs. Our regression analysis further supports this finding, where gastritis-related diseases reduce the likelihood of prescribing PPIs by 8–11% (Table 5). This observation is consistent with a study conducted in an ambulatory care setting in the United States, where more than half of PPI users did not have documented gastrointestinal diagnoses [38]. Similarly, research published by a group in Qatar showed that 78% of PPI users lacked specific indications for their use [39]. It is paramount to point out that our study is retrospective in nature and is subject to limitations, particularly regarding the risk of incomplete medical history during data collection. To address this issue, the Rx–risk tool was applied to identify the unreported comorbidities and patterns of medication prescribing. In addition, there were no significant differences in the prevalence of NSAIDs use between PPI users and non–users, suggesting a potential underutilization of PPI for appropriate indications. These findings emphasize the need to improve PPI prescribing practices and revise the regional guidelines for PPI prescribing in order to enhance their safety and appropriate utilization.

In conjunction with other results, PPI users in the current study were regularly prescribed concurrent medications known to induce gastro-esophageal reflux diseases, such as NSAIDs or CCBs, and medications that may aggravate GI bleeding, including antiplatelet and anticoagulant drugs [35,37,40]. NSAIDs were the most commonly co-prescribed medications among PPI users (48% were prescribed NSAIDs) and were associated with PPI use among middle-aged patients (OR = 1.1). Similar findings were reported by Madi et al., where NSAIDs were found to be the most co-prescribed medications with PPI [39]. However, this association did not reach statistical significance in a univariate analysis when compared to non-PPI users, who also showed similar rates of NSAID usage, indicating inconsistent prescribing patterns and PPI utilization among NSAID users. The American College of Gastroenterology (ACG) guidelines recommend initiating PPI therapy for patients using antiplatelet medications and at increased risk of gastric bleeding [41]. PPI users in this study presented a higher prevalence of antiplatelet medication prescribing (13%) compared to non-users, whereas the regression analysis revealed that concurrent intake of antiplatelet medications increases the likelihood of having PPI therapy by 8–10%. Consequently, our findings suggest that an appropriate tapering of PPI medication and the incorporation of lifestyle modification are warranted, owing to the lack of established guidelines for long-term PPI use alongside medications intended for lifelong therapy.

Upon dividing the entire cohort receiving PPI into two groups, HD and MD users, we found that HD PPI users were older, prescribed more medications, and had high CCI levels. This pattern aligns with findings from previous studies conducted in Saudi Arabia and other countries [35,36,37]. Our results emphasize the need for an evidence-based review of the current PPI prescribing pattern, as the use of HD PPIs appears to be associated with various safety issues, including inappropriate prescription patterns and an increased risk of ADRs linked to their long-term use [34].

Consistent with findings from other studies, females were more likely to receive prescriptions for PPIs compared to males [36,37], even though these gender-based differences were not observed in other studies [39,42]. One possible explanation is that females generally seek healthcare services more frequently for their health concerns compared to males [43]. This finding is crucial for the care of older female patients, as inappropriate PPI prescribing, whether MD or HD, for long-term use, has been linked to an increased risk of ADRs such as osteoporosis [24]. Considering the peri- and postmenopausal changes experienced by older females, placing them at an already higher risk of developing osteoporosis, PPI use may magnify their potential risk to the detrimental loss of bone density [44]. As a consequence, older female patients are at greater risk of bone fractures that could substantially impact their quality of life.

Consistent with previous findings [35,36,37,39], omeprazole and esomeprazole emerged as the most commonly prescribed PPIs in this study. Regarding clinically significant DDIs, the pro-drug, clopidogrel, has been reported to interact with the mentioned PPI drugs, potentially reducing its antiplatelet effects by reducing plasma levels of activated clopidogrel [41]. Given that omeprazole inhibits CYP3A4 and esomeprazole inhibits CYP2C19, the possibility of DDIs should be considered and the selection of PPIs should be reviewed to avoid any adverse clinical outcomes [45]. On the other hand, according to FDA recommendations, the use of omeprazole or esomeprazole should be avoided in patients taking clopidogrel due to an increased risk of bleeding [46]. The pattern of concomitant prescribing of both medications revealed in our study should not be interpreted as indicative of malpractice. The cross-sectional design of the study limits our ability to assess the impact of combining antiplatelets with PPIs on cardiovascular-adverse events, such as recurrent cardiac events and changes in the coagulation profile. Furthermore, a recent study that investigated the clinical outcomes of concomitant use of PPIs and dual antiplatelet therapy reported no clear evidence linking PPI use with adverse cardiovascular events [47].

A notable strength of this study is that it is the first investigation into the pattern of PPI prescribing, and factors contributing to their pattern of use and the risk of ADRs, in the Eastern Region of Saudi Arabia. The study’s robustness and reliability are enhanced by its large cohort of patients (*n* = 41,084) providing substantial data for analysis.

This study emphasizes the ongoing challenges associated with excessive PPI prescribing. A recent study by Asdaq et al. showed that health providers in Saudi Arabia recognize the overuse of PPIs, with 87.6% of physicians and 93.4% of pharmacists supporting widespread educational interventions promoting the rational use of PPIs among medical professionals and the public [13]. The referenced study also revealed that many physicians routinely prescribe PPIs for symptoms such as nausea, vomiting, and flatulence. Moreover, it highlighted the drawbacks of PPI use in the community, a topic outside the scope of our study. It is important to note that the results of the study are based on a different regional population of Saudi Arabia (Riyadh), with the demographic characteristics of participants distinctly different from those in our current study. This finding, in itself, highlights exciting facets of consideration for future discussion and research.

However, several limitations should be acknowledged in this study. Firstly, the duration of PPI use could not be feasibly determined as data were collected retrospectively, which limited the ability to evaluate the appropriateness of PPI use and its efficacy. Secondly, the absence of follow-up data prevented the assessment of longer-term treatment outcomes associated with PPIs, including their effectiveness and self-reported ADRs. Due to the retrospective study design, the accuracy and robustness of the data rely on the quality of existing records; thus, issues such as missing and incomplete data, and other confounding factors, should be considered. To alleviate these challenges, various tools such as Rx–risk and CCI were utilized. Furthermore, binary logistical regression and multi-collinearity using the variance inflation factor were used to address uncertainty arising from confounding factors. Finally, this study was cross-sectional in nature; thus, it was not possible to describe the trajectory of PPI use over time or how this related to changes in the disease progression and management trajectories.

It is crucial to clarify that the results of this study should not be interpreted as implying inappropriate use of PPIs. There were many valid indications for prescribing these medications, such as their use with NSAIDs and for treatment of gastritis-related diseases. However, similar indications were also observed among PPI non-users, raising concerns about missing therapeutic opportunities. The present study has illuminated the persistent issue of overutilization of PPIs within this patient cohort.

For future practice, it is essential to provide valid justification and reasoning for prescribing PPIs, in order to blunt the plausible risk of ADRs associated with their inappropriate use. There is a need for reassessment of our current practices to determine the necessity of PPI therapy. Educational interventions and institutional prescribing guidelines are recommended to further reduce inappropriate use of PPIs in clinical practice. Future research should focus on investigating the duration of PPI use, and the prevalence of long-term ADRs associated with their use, establishing a comprehensive assessment of PPI appropriateness and their impact on patient health outcomes. Moreover, a longitudinal study could be designed to examine the initial duration of PPI prescriptions and the frequency of therapy refills without adequate review, supporting pharmacist interventions in pharmacist-led clinics. A survey-based study to investigate the availability of pantoprazole in all hospitals for patients concurrently taking clopidogrel would also shed light on critical perceptions. Lastly, a study examining patients’ experiences during PPI withdrawal could provide valuable insights into the challenges and outcomes associated with discontinuing PPI therapy. Addressing these research queries to better understand the nature of PPI utilization and its impact will ultimately lead to improved prescribing practices and enhanced patient care.

## 5. Conclusions

Despite the acknowledged limitations, the risk of ADRs associated with PPI prescribing patterns should be assessed for effective therapeutic practices, particularly among older patients or those receiving high-intensity doses that may increase their susceptibility to ADRs. It is worth noting that gastritis-related diseases were more prevalent among PPI non-users compared to users, even though both groups were equally prescribed NSAIDs and CCBs. Ultimately, the findings of this study highlight the importance of re-evaluating PPI prescription in clinical practice. Comprehensive medication reviews are essential to support appropriate PPI prescribing to ensure optimal efficacy with minimum ADRs.

## Figures and Tables

**Table 1:** Definition of low-, medium-, and high-dose-intensity proton pump inhibitors utilized in this study.

	Low Dose	Medium Dose	High Dose
Omeprazole	≤10 mg	20 mg	>20 mg
Esomeprazole	Not applicable	20 mg	>20 mg
Pantoprazole	≤20 mg	40 mg	>40 mg

**Table 2:** Characteristics of the included patients categorized based on proton pump inhibitor utilization.

Characteristic	Entire Cohort	PPI Users	PPI Non-Users	*p*-Value
	*n* = 41,084	*n* = 12,812	*n* = 28,272	
Age, median (IQR)	52 (42–73)	52 (42–73)	52 (42–73)	0.08
Gender (Female), *n* (%)	20,800 (50.6)	6603 (51.5)	14,197 (50.2)	0.013
Weight, mean (SD)	75.1 (17.3)	74.4 (18)	75.4 (16.9)	<0.001
CCI, mean (SD)	2.3 (2.2)	2.2 (2.1)	2.3 (2.2)	0.003
NPM, mean (SD)	6.5 (5.6)	5.7 (5.2)	6.9 (5.8)	<0.001
CrCl, mean (SD)	98.7 (42)	98.6 (45)	98.7 (41)	<0.001
Reported Comorbidities				
Dementia, *n* (%)	55 (0.1)	15 (0.1)	40 (0.1)	0.5
Depression, *n* (%)	1599 (4)	466 (4)	1133 (4)	0.07
Pain, *n* (%)	25,257 (62)	7794 (61)	17,463 (62)	0.07
Gastritis-related disease, *n* (%)	13,563 (33)	4061 (32)	9502 (34)	<0.001
Constipation, *n* (%)	3842 (9)	1134 (9)	2708 (10)	0.02
Renal Disease, *n* (%)	600 (2)	190 (2)	410 (2)	0.8
Anemia, *n* (%)	10,025 (24)	3085 (24)	6940 (25)	0.3
Respiratory disorder, *n* (%)	3586 (9)	1134 (9)	2452 (9)	0.6
Arthritis-related disease, *n* (%)	29,012 (71)	9018 (70)	19,994 (71)	0.5
Osteoporosis, *n* (%)	7912 (19)	2430 (19)	5482 (19)	0.3
Co-prescribed Medications				
Antacid, *n* (%)	2241 (6)	689 (5)	1552 (6)	0.6
Anticoagulants, *n* (%)	4646 (11)	1514 (12)	3132 (11)	0.03
Antiplatelets, *n* (%)	5036 (12)	1655 (13)	3381 (12)	0.006
CCB, *n* (%)	3997 (10)	1327 (10)	2670 (9)	0.004
Nitrate, *n* (%)	443 (1)	154 (1)	289 (1)	0.1
NSAID, *n* (%)	19,504 (48)	6135 (48)	13,369 (47)	0.3
TCA, *n* (%)	1375 (3)	436 (3)	939 (3)	0.7
SSRI, *n* (%)	335 (1)	108 (1)	227 (1)	0.7
Paracetamol, *n* (%)	24,163 (59)	7507 (59)	16,656 (59)	0.5

PPI: Proton pump inhibitor, IQR: Inter-quartile range, SD: Standard range, CCI: Charlson comorbidity index, NPM: Number of prescribed medications, CrCl: Creatinine clearance. CCB: Calcium channel blocker, NSAID: Non-steroidal anti-inflammatory drugs, TCA: Tricyclic anti-depressant, SSRI: Selective serotonin reuptake inhibitor.

**Table 3:** Characteristic of the proton pump inhibitor users categorized based on dose intensity.

Characteristic	PPI Users	Medium-Dose Users	High-Dose Users	*p*-Value
	*n* = 12,812	*n* = 8683	*n* = 4129	
Age, median (IQR)	52 (42–73)	52 (41–73)	53 (42–71)	<0.001
Gender (Female), *n* (%)	6603 (52)	4828 (56)	1775 (43)	0.013
Weight, mean (SD)	74 (18)	71 (21)	81 (6)	<0.001
CCI, mean (SD)	2.2 (2.1)	2.2 (2)	2.3 (2.1)	0.003
NPM, mean (SD)	6 (5.2)	3 (1.5)	12 (4.7)	<0.001
CrCl, mean (SD)	99	96 (47)	105 (40)	<0.001
Reported Comorbidities				
Dementia, *n* (%)	15 (0.1)	11 (0.1)	4 (0.1)	0.7
Depression, *n* (%)	466 (4)	318 (4)	148 (4)	0.8
Pain, *n* (%)	7794 (61)	5271 (61)	2523 (61)	0.7
Gastritis related disease, *n* (%)	4061 (32)	2705 (31)	1356 (33)	0.06
Constipation, *n* (%)	1134 (9)	785 (9)	349 (9)	0.3
Renal Disease, *n* (%)	190 (2)	144 (2)	46 (1)	0.02
Anemia, *n* (%)	3085 (24)	2104 (24)	981 (24)	0.6
Respiratory disorder, *n* (%)	1134 (8)	753 (9)	381 (9.)	0.3
Arthritis related disease, *n* (%)	9018 (70)	6174 (71)	2844 (69)	0.01
Osteoporosis, *n* (%)	2430 (19)	1629 (19)	801 (19)	0.4
Co-prescribed Medications				
Antacid, *n* (%)	689 (5)	499 (6)	190 (5)	0.007
Anticoagulants, *n* (%)	1514 (12)	1066 (12)	448 (11)	0.02
Antiplatelets, *n* (%)	1655 (13)	1179 (14)	476 (12)	0.001
CCB, *n* (%)	1327 (10)	985 (11)	342 (8)	<0.001
Nitrate, *n* (%)	154 (1)	116 (1)	38 (1)	0.04
NSAID, *n* (%)	6135 (48)	4215 (49)	1920 (47)	0.03
TCA, *n* (%)	436 (3)	316 (4)	120 (3)	0.03
SSRI, *n* (%)	108 (1)	84 (1)	24 (0.6)	0.03
Paracetamol, *n* (%)	7507 (59)	5180 (60)	2327 (56)	<0.001

PPI: Proton pump inhibitor, IQR: Inter-quartile range, SD: Standard range, CCI: Charlson comorbidity index, NPM: Number of prescribed medications, CrCl: Creatinine clearance. CCB: Calcium channel blocker, NSAID: Non-steroidal anti-inflammatory drugs, TCA: Tricyclic anti-depressant, SSRI: Selective serotonin reuptake inhibitor.

**Table 4:** Characteristic of the proton pump inhibitor users categorized by age groups: middle-aged versus older patients.

Characteristic	PPI Users	Middle-Aged Patients	Older Patients	*p*-Value
	*n* = 12,812	*n* = 10,228	*n* = 2584	
Age, median (IQR)	52 (42–73)	49 (41–60)	72 (66–87)	<0.001
Gender (Female), *n* (%)	6603 (52)	5188 (51)	1415 (55)	<0.001
Weight, mean (SD)	74 (18)	75 (18)	72 (16)	<0.001
CCI, mean (SD)	2 (2)	2 (2)	5 (2)	<0.001
NPM, mean (SD)	6 (5)	6 (5)	6 (5)	0.09
CrCl, mean (SD)	99 (45)	105 (45)	74 (32)	<0.001
PPI Prescribing				
Omeprazole, *n* (%)	7252 (57)	5771 (56)	1481 (57)	0.4
Esomeprazole, *n* (%)	5521 (43)	4424 (43)	1097 (43)	0.5
Pantoprazole, *n* (%)	39 (0.3)	33 (0.3)	6 (0.2)	0.5
Medium dose intensity, *n* (%)	8683 (68)	6940 (68)	1743 (68)	0.7
Reported Comorbidities				
Dementia, *n* (%)	15 (0.1)	2 (0.01)	13 (0.5)	<0.001
Depression, *n* (%)	466 (4)	365 (4)	101 (4)	0.4
Pain, *n* (%)	7794 (61)	6280 (61)	1514 (59)	0.009
Gastritis related disease, *n* (%)	4061 (32)	2979 (29)	1082 (42)	<0.001
Constipation, *n* (%)	1134 (9)	747 (7)	387 (15)	<0.001
Renal Disease, *n* (%)	190 (2)	111 (1)	79 (3)	<0.001
Anemia, *n* (%)	3085 (24)	2430 (24)	655 (25)	0.09
Respiratory disorder, *n* (%)	1134 (9)	796 (8)	338 (13)	<0.001
Arthritis related disease, *n* (%)	9018 (70)	7244 (71)	1774 (69)	0.03
Osteoporosis, *n* (%)	2430 (19)	1806 (18)	624 (24)	<0.001
Co-prescribed Medications				
Antacid, *n* (%)	689 (5)	566 (6)	123 (5)	0.1
Anticoagulants, *n* (%)	1514 (12)	1245 (12)	269 (11)	0.01
Antiplatelets, *n* (%)	1655 (13)	1385 (14)	270 (11)	<0.001
CCB, *n* (%)	1327 (10)	1104 (11)	223 (9)	0.001
Nitrate, *n* (%)	154 (1)	129 (1)	25 (1)	0.2
NSAID, *n* (%)	6135 (48)	4936 (48)	1199 (46)	0.09
TCA, *n* (%)	436 (3)	365 (4)	71 (3)	0.04
SSRI, *n* (%)	108 (1)	79 (1)	29 (1)	0.08
Paracetamol, *n* (%)	7507 (59)	6093 (60)	1414 (55)	<0.001

PPI: Proton pump inhibitor, IQR: Inter-quartile range, SD: Standard range, CCI: Charlson comorbidity index, NPM: Number of prescribed medications, CrCl: Creatinine clearance. CCB: Calcium channel blocker, NSAID: Non-steroidal anti-inflammatory drugs, TCA: Tricyclic anti-depressant, SSRI: Selective serotonin reuptake inhibitor.

**Table 5:** Factors associated with proton pump inhibitors prescribing across the entire cohort, older patients, and middle-aged patients.

	Entire Cohort		Older Patients		Middle-Aged	
	Odd Ratio	95% CI	Odd Ratio	95% CI	Odd Ratio	95% CI
Age	1	0.99–1.1	1.001	0.99–1.01	1	0.99–1.01
Gender (Female)	0.98	0.93–1.03	0.92	0.83–1.02	1	0.95–1.05
weight	1	0.99–1	0.998	0.99–1.01	1	0.99–1.01
CCI	0.99	0.98–1.01	0.99	0.97–1.02	1	0.98–1
NPM	0.96	0.95–0.97	0.97	0.96–0.98	0.96	0.955–0.964
CrCl	1	0.99–1.01	1	0.99–1.01	1.1	0.99–1.01
Comorbidities						
Dementia	0.84	0.46–1.5	0.8	0.43–1.5	1.2	0.2–6.7
Depression	0.91	0.82–1.02	0.9	0.7–1.01	0.92	0.8–1.05
Pain	0.96	0.92–1.01	0.94	0.85–1.01	0.97	0.92–1.02
Gastritis realted disease	0.92	0.87–0.97	0.9	0.8–1.01	0.92	0.87–0.98
Constipation	0.93	0.87–1.01	0.95	0.8–1.01	0.92	0.84–1.01
Renal Disease	1.1	0.9–1.3	1.05	0.8–1.4	1.1	0.87–1.4
Anaemia	0.98	0.92–1.03	0.99	0.9–1.1	0.98	0.92–1.03
Arthritis related disease	0.98	0.94–1.03	1.06	0.96–1.2	0.96	0.91–1.01
Osteoporosis	0.99	0.93–1.05	0.85	0.75–0.96	1.04	0.97–1.1
Medications						
Antacid	0.97	0.88–1.06	0.9	0.75–1.2	0.98	0.9–1.1
Anticoagulant	1.06	0.99–1.1	1.1	0.93–1.3	1.06	0.98–1.14
Antiplatelet	1.08	1.01–1.15	0.97	0.8–1.1	1.1	1.03– 1.2
CCB	1.09	1.01–1.16	1.1	0.9–1.3	1.09	1.01–1.2
NSAID	1.02	0.98–1.06	1.1	1.01–1.2	1.001	0.96–1.05
TCA	1	0.9–1.1	0.9	0.8–1.2	1.03	0.91–1.17
SSRI	1.03	0.82–1.3	1.4	0.9–2.2	0.94	0.7–1.2
Paracetamol	0.98	0.94–1.02	0.99	0.9–1.1	0.98	0.93–1.02

CI: Confidence Interval, NPM: Number of prescribed medications, CCI: Charlson comorbidity index, CrCl: Creatinine clearance SSRI: Selective serotonin reuptake inhibitors, TCA: Tricyclic antidepressants, NSAID: Non-steroidal anti-inflammatory drugs, CCB: Calcium Channel blockers.

**Table 6:** Factors associated with medium or high intensity dose of proton pump inhibitors prescribing across the entire cohort, older patients, and middle-aged patients.

	Entire Cohort		Older Patients		Middle-Aged Patients	
	Medium Dose	High Dose	Medium Dose	High Dose	Medium Dose	High Dose
Age	1 (0.99–1.01)	1 (0.99–1.01)	1 (0.99–1.01)	0.98 (0.97–0.99)	1 (0.99–1.01)	1 (0.99–1.02)
Gender (Female)	1 (0.95–1.06)	0.85 (0.8–0.9)	1.1 (0.93–1.2)	0.8 (0.7–0.99)	1 (0.9–1.1)	0.85 (0.78–0.92)
weight	0.992 (0.995–0.99)	1 (0.99–1.01)	0.992 (0.987–0.996)	1.01 (1.004–1.02)	0.992 (0.99–0.994)	1 (0.99–1.01)
CCI	0.99 (0.97–1.01)	1 (0.99–1.02)	1 (0.96–1.02)	1 (0.97–1.1)	1 (0.97–1.01)	1 (0.97–1.02)
NPM	0.7 (0.69–0.71)	1.13 (1.12–1.14)	0.7 (0.68–0.72)	1.13 (1.11–1.14)	0.705 (0.7–0.71)	1.13 (1.127–1.14)
CrCl	1.002 (1.001–1.003)	0.998 (0.997–0.999)	1.004 (1.001–1.006)	1.2 (1.02–1.4)	1.001 (1.001–1.002)	0.998 (0.997–0.999)
Comorbidities						
Dementia	0.9 (0.4–1.8)	0.9 (0.3–2.5)	0.9 (0.4–1.8)	1 (0.3–2.5)	0.9 (0.4–1.8)	0.9 (0.3–2.5)
Depression	1 (0.9–1.2)	0.92 (0.768–1.1)	1 (0.7–1.3)	1 (0.7–1.5)	1 (0.9–1.2)	0.9 (0.8–1.1)
Musculoskeletal pain	0.97 (0.9–1.02)	0.98 (0.91–1.05)	0.9 (0.8–0.98)	1 (0.9–1.2)	0.97 (0.9–1.02)	1 (0.9–1.1)
Gastritis related disease	0.95 (0.89–1.01)	0.95 (0.87–1.04)	0.9 (0.8–1.02)	1 (0.85–1.2)	0.95 (0.9–1.01)	1 (0.9–1.04)
Constipation	1 (0.9–1.1)	0.85 (0.75–0.97)	0.98 (0.9–1.1)	0.9 (0.7–1.1)	1 (0.9–1.09)	0.85 (0.75–0.97)
Renal Disease	1.2 (0.98–1.5)	0.7 (0.5–0.99)	1.3 (0.9–1.8)	0.6 (0.3–0.99)	1.2 (0.98–1.5)	0.7 (0.5–0.99)
Anaemia	1 (0.9–1.04)	1 (0.9–1.1)	1 (0.9–1.1)	1. (0.9–1.2)	1 (0.91–1.04)	1 (0.9–1.1)
Arthritis related disease	1 (0.94–1.1)	0.9 (0.8–0.98)	1.1 (0.99–1.3)	1 (0.8–1.1)	1 (0.94–1.05)	0.9 (0.8–0.98)
Osteoporosis	0.95 (0.9–1.02)	1.1 (0.96–1.2)	0.9 (0.73- 0.99)	0.8 (0.6–0.95)	1 (0.88–1.02)	1 (0.9–1.2)
Medications						
Antacid	1 (0.9–1.1)	0.8 (0.7–0.98)	1 (0.8–1.3)	0.7 (0.5–1.1)	1 (0.86–1.08)	0.8 (0.7–0.97)
Anticoagulant	1 (0.9–1.1)	1 (0.9–1.1)	1.5 (0.9–1.3)	1 (0.8–1.3)	1 (0.92–1.08)	0.97 (0.9–1.1)
Antiplatelet	1 (0.9–1.1)	0.95 (0.85–1.1)	1.1 (0.9–1.3)	0.7 (0.5–0.9)	1 (0.95–1.11)	0.95 (0.9–1.1)
CCB	1.1 (0.99–1.2)	0.85 (0.75–0.96)	1.2 (0.9–1.4)	0.7 (0.5–0.97)	1 (0.98–1.2)	0.9 (0.8–0.96)
NSAID	1 (0.97–1.1)	0.95 (0.9–1.03)	1.1 (0.99–1.3)	1.1 (0.9–1.3)	1 (0.94–1.05)	1 (0.9–1.03)
TCA	0.97 (0.8–1.1)	0.9 (0.7–1.1)	0.9 (0.7–1.3)	0.7 (0.4–1.2)	1 (0.84–1.1)	1 (0.7–1.1)
SSRI	1.1 (0.9–1.5)	0.7 (0.5–1.5)	2 (1.2–3.7)	0.6 (0.2–1.6)	1 (0.85–1.5)	1 (0.5–1.2)
Paracetamol	0.99 (0.9–1.04)	0.9 (0.8–0.97)	1.1 (0.9–1.2)	0.9 (0.7–0.99)	1 (0.84–1.04)	0.9 (0.8–0.96)

Results represent odds ratio (95% confidence interval) generated from multinominal logistic regression with reference to no proton pump inhibitor use. NPM: Number of prescribed medications, CCI: Charlson comorbidity index, CrCl: Creatinine clearance, SSRI: Selective serotonin reuptake inhibitors, TCA: Tricyclic antidepressants, NSAID: Non-steroidal anti-inflammatory drugs, and CCB: Calcium Channel blockers.

## Data Availability

The datasets analyzed during the current study will be made available on reasonable request. Data will be made available for scientific purposes for researchers whose proposed use of the data has been approved by the research team.
